# 

**Published:** 1890-11

**Authors:** 


### CONTENTS.

*EDITORIALS.* PAGE

Medicinal Aggravation, . . . . . . . . . . . . . 481
The Teaching of Pure Homœopathy in the Colleges, . . . 483

*MISCELLANEOUS.*

Some Results from the Abuse of Quinine. J. T. Martin, M. D., . 484
An Autograph Letter of Hahnemann, . . . . . . . . 492
An Autograph Letter of Hahnemann, . . . . . . . . 493
The Insanity of Jealousy. S. L., . . . . . . . . . 494
The Routine Treatment of Fresh Gonorrhœa and Straight Homœopathy. Thomas Skinner, M. D., . . . . . . . . . . 498
A Funny Symptom—Sulphur $55^{m}$. M. A. A. Wolff, M. D., . . 501
A Materia Medica Pura. E. J. L., . . . . . . . . . 504
Erysipelas. G. M. Pease, M. D., . . . . . . . . . 505
British Medicinal Plants. Alfred Heath, M. D., F. L. S , . . 508
The Relation of Drugs to Pregnancy. L. Hamilton Evans, M. D., . 510
Syphilis and Gonorrhœa. I. Dever, M. D., . . . . . . 513
Homœopathy and Pathology. B. L. B. Baylies, M. D., . . . 515
Clinical Cases. Edward Rushmore, M. D., . . . . . . 516
Clinical Cases. Clarence N. Payne, M. D., . . . . . . 518
Grafts. W. A. Yingling, M. D., . . . . . . . . . 520
Verifications in the October Number. Wm. Jefferson Guernsey, M. D., . . . . . . . . . . . . . . 522
Weakness after Urination. Robert Farley, M. D., . . . . . 522
A Proving of Nux Vomica. Ella M. Tuttle, M. D., . . . . 523

*BOOK NOTICES.*

Census Bulletin No. 9, . . . . . . . . . . . . 524
" " " 6, . . . . . . . . . . . . 524
" " " 10, . . . . . . . . . . . . 524
" " " 11, . . . . . . . . . . . . 524
Journal of Balneology, . . . . . . . . . . . . 524
Physical Diagnosis and Practical Urinalysis, . . . . . . 525
The Baltimore Family Health Journal, . . . . . . . . 525
The Family Homœopathist, . . . . . . . . . . . 526
Epilepsy; Its Pathology and Treatment, . . . . . . . 526
Kate Field's Washington, . . . . . . . . . . . 527
Report of International American Conference, . . . . . . 527
Report on Sanitary and Quarantine Regulations, . . . . . 527

*NOTES AND NOTICES.*

Grace Hospital—Removals—Fun for Doctors, . . . . . . 528

